# Gastrointestinal Cell Mediated Immunity and the Microsporidia

**DOI:** 10.1371/journal.ppat.1002775

**Published:** 2012-07-12

**Authors:** Magali M. Moretto, Imtiaz A. Khan, Louis M. Weiss

**Affiliations:** 1 Department of Microbiology, Immunology and Tropical Medicine, George Washington University, Washington, D.C., United States of America; 2 Departments of Medicine and Pathology, Albert Einstein College of Medicine, Bronx, New York, United States of America; University of Wisconsin Medical School, United States of America

## Introduction and Basic Biology

The microsporidia are obligate intracellular parasites that were previously thought to be “primitive” eukaryotes, but which are now recognized to be either related to or a sister group to the Fungi [1]. These protists are relatively common enteric pathogens that usually result in self-limited or asymptomatic infections in humans. Microsporidia were initially recognized as pathogens causing pébrine in silkworms in the late 19th century by Louis Pasteur. The cause of pébrine was named *Nosema bombycis*, providing the initial organism in the phylum Microsporidia that now includes approximately 170 genera containing over 1,400 species, many of which are of medical, veterinary, and agricultural importance [2]. All of the microsporidia form characteristic spores containing a specialized invasion organelle consisting of an internal polar tube that is attached to the inside of the anterior end of the spore by an anchoring disc and coils around the sporoplasm in the spore. During germination, the polar tube rapidly everts, forming a hollow tube that brings the sporoplasm into intimate contact with the host cell. The polar tube provides a bridge to deliver the sporoplasm to the host cell. The mechanism by which the polar tube interacts with the host cell membrane is not fully known, but this may require the participation of the host cell. Glycosylation is probably important in polar tube structure and function. This is supported by studies demonstrating carbohydrate residues on intact polar tubes, e.g., concanavalin A binds to the polar tube of several different microsporidia and there is biochemical evidence that major polar tube protein (PTP1) has O-linked mannosylation [3]. Pretreatment of host cells with mannose decreased infection by *Encephalitozoon hellem* consistent with an interaction between polar tube mannosylation and some unknown host cell mannose-binding molecule [3]. The spore coat consists of an electron-dense, proteinaceous exospore, an electron-lucent endospore composed of chitin and protein, and an inner membrane or plasmalemma. Several spore wall proteins are also modified by post-translational glycosylation involving mannosylation (L. Weiss, unpublished data). These modifications are likely important in adherence of the spore wall to mucin or to host cells during passage of the spores in the gastrointestinal tract, facilitating invasion; e.g., exogenous glycosaminoglycans decrease the adherence of spores to a host cell monolayer [4]. Spores are resistant to environmental conditions and this allows these organisms to persist in the environment, facilitating their transmission between hosts. These resistant spores may also allow these organisms to persist in infected tissues.

The genome size of microsporidia varies from 2.3 to 19.5 Mb [2], with *Encephalitozoon cuniculi* being 2.9 Mb, *E. hellem* 2.5 Mb, and *Encephalitozoon intestinalis* 2.3 Mb, which are among the smallest eukaryotic nuclear genomes identified. These organisms are probably diploid, there are almost no introns in these genomes, the gene density is high, and proteins are shorter than corresponding yeast genes. These intracellular pathogens have lost many metabolic pathways, becoming dependent on their host cells [5]. A collaboration between Patrick Keeling (University of British Columbia), Saul Tzipori (Tufts University), Elizabeth Didier (Tulane University), Louis M. Weiss (Albert Einstein), and the Broad Institute (MIT) has resulted in the sequencing of the genomes of *Enterocytozoon bieneusi*, *E. intestinalis*, *E. cuniculi* (types 1, 2, and 3), and *E. hellem*, providing genomic data on these human pathogenic microsporidia [5]. In addition, the genome of *Anncaliia (Brachiola) algerae* is currently undergoing genome annotation by this same collaborative group [6]. Other groups have completed the genomes of several insect and invertebrate microsporidian pathogens, including *Nosema ceranae, Octosporea bayeri* and *Vavria culicis floridensis*, *Nosema bombycis*, and the soil nematode pathogen *Nematocida parisii*. Genome data on these various microsporidia is being made available at EuPathDB under MicrosporidiaDB (http://microsporidiadb.org/micro/).

## The Pathogenic Potential of the Microsporidia

Before the AIDS pandemic, the microsporidia were recognized primarily as pathogens of a variety of animals of agricultural importance (e.g., fish, fur bearing animals, and beneficial and pestilent insects) and animals used in laboratory research (e.g., rabbits, rodents, and primates). Microsporidia were used as biological control agents of insect pests, the most notable example being *Antonospora locustae* for the control of grasshoppers. There were case reports of human infection, but their infrequency relegated these pathogens essentially to the status of medical curiosity. Since the mid-1980s, however, microsporidia have garnered increasing medical attention, with over 400 cases associated with AIDS reported in the literature by 1994 and diagnostic surveys demonstrating infection rates as high as 70% in HIV-infected populations [2]. In patients with AIDS, especially in those with ≤100 CD4+ cells/mm^3^, the most common clinical manifestation of microsporidiosis is chronic diarrhea and wasting due to enteric infection, but the spectrum of disease due to these pathogens is broad and includes hepatitis, peritonitis, keratoconjunctivitis, sinusitis, bronchitis, pneumonia, cystitis, nephritis, myositis, encephalitis and other cerebral infections, urethritis, prostatitis, oral ulcers, osteomyelitis, and cellulitis [2]. Microsporidia also cause infections in other immune-suppressed individuals (reviewed in [7]). Since the 1990s, over 30 cases of microsporidiosis have been described in solid organ and bone marrow transplant recipients, most of whom received some form of immune suppressive therapy to ensure graft survival; several of these infections were life-threatening and resulted in fatal outcomes (reviewed in [8]). While in most cases it was unclear whether these infections originated from the donor or from latent infection of the recipient, the inability of either to have completely cleared the parasite despite initially displaying no clinical signs of infection suggests that the microsporidia are capable of modulating the immune response of the host in favor of their own survival.

To date, at least 14 identified and two indeterminate species of Microsporidia have been found to infect humans, representing nine genera including *Microsporidium* (a taxon reserved for species of indeterminate assignment). The following microsporidian phyla have been demonstrated to cause human infection [2]: *Nosema* (*N. corneum* renamed *Vittaforma corneae* and *N. algerae* reclassified initially as *Brachiola algerae* and now as *Anncaliia algerae*), *Pleistophora*, *Encephalitozoon*, *Enterocytozoon*, *Septata* (reclassified as *Encephalitozoon*), *Trachipleistophora, Anncaliia*, *Brachiola* (reclassified as *Anncaliia*), *Vittaforma, Tubulinosema* [9], and *Microsporidium*.

Now widely acknowledged as opportunistic pathogens, evidence has emerged that the microsporidia cause disease in immune-competent individuals as well. Cases of microsporidiosis have been identified from all continents except Antarctica [2]. Surveys of pathogens seen in stool samples in Africa, Asia, South America, and Central America have demonstrated that microsporidia are often found during careful stool examinations. Notable examples of microsporidiosis in immune-competent humans include gastrointestinal infections that have been discovered in travelers to and residents of underdeveloped countries, and ocular infections in contact lens wearers. Indeed, the high seroprevalence of anti-microsporidian antibodies revealed by surveys of immune-competent individuals suggests that microsporidiosis in the general population may be common but self-limiting or asymptomatic. Despite the growing significance of the microsporidia, relatively little is known about environmental reservoirs for these pathogens, and modes of transmission to humans have not been explicitly documented. However, there is evidence that infections can occur by multiple routes (enumerated in [2]) including waterborne, respiratory, sexual, congenital, zoonotic transmission, and in ocular infection by traumatic inoculation into the cornea. Human infection with *A. algerae*, a microsporidium initially identified from mosquitoes, suggests that vector-borne transmission may also occur [10]. Microsporidian spores are commonly found in surface water, and human pathogenic microsporidia have been found in municipal water supplies, tertiary sewage effluent, and ground water [11], [12]. Because of the probable risk of environmental transmission, the U.S. Environmental Protection Agency included these organisms on the most recent Candidate Contaminant Lists CCL-1 and -2 in 1998 and 2005, respectively; these actions identify the microsporidia as pathogens that may require regulation under the Safe Drinking Water Act. The ubiquity and hardiness of the spore ensure that much of the human population is at risk for infection.

## Gastrointestinal Immune Response to the Microsporidia

Studies on mammalian microsporidiosis usually utilize *E. cuniculi*, as it has a large host range, can cause natural infections in laboratory animals (e.g., mice, rats, and rabbits), and can be grown in tissue culture [13]. Experimental murine infections with *E. cuniculi* mimic human infection, thereby supporting studies that use this model to dissect the immune response to microsporidiosis [14]. Although protection to microsporidia is predominantly dependent on the initiation of a strong T cells response [15], [16], dendritic cells (DC) play a critical role in priming of the T cell response via recognition of the pattern recognition receptors (PRRs) expressed by the pathogens [17]. This phenomenon involves the pathogen-associated molecular patterns (PAMPs) including an array of toll-like receptors (TLRs) expressed on the surface of the cell. Moretto and Khan reported that optimal CD8 T cell response to microsporidia was mediated by TLR4 expression by murine DC [18]; however, a role for TLR2 has also been described in the activation of human primary macrophages during microsporidiosis [19].

**Figure 1:**
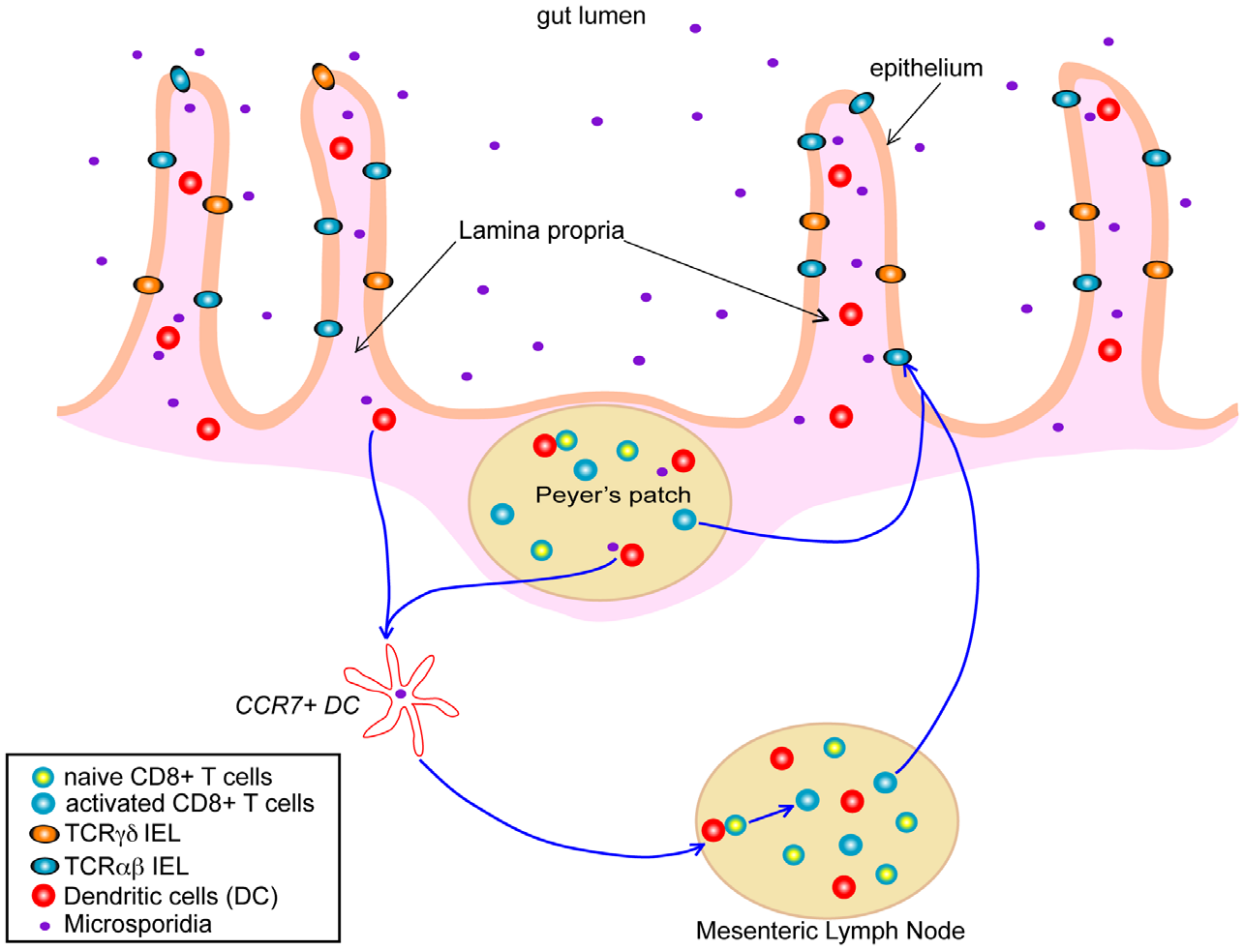
Putative model of the development of antigen-specific IEL response in the gut after infection with microsporidia. Once the infecting microsporidia cross the gut intestinal barrier, they are picked up by dendritic cells (DC) either in the Peyer's patch or the lamina propria. These DC become activated, express CCR7, and then migrate to the Mesenteric Lymph Nodes (MLN), where they encounter naïve CD8^+^ T cells. After antigen presentation, these CD8^+^ T cells become activated and now express different homing and chemokine receptors that allow them to traffic back to the gut mucosa, where they disseminate throughout the intestinal epithelium, becoming TCRαβIEL and TCRγδIEL.

Systemic infection, via the peritoneal route, with *E. cuniculi* induces protective immunity that is highly dependent on a cytotoxic CD8^+^ T cells response, with the CD4^+^ subset playing a minor role in the immune response [20], [21]. The situation is different when investigating the per-oral (i.e., the natural route) infection, where both the CD4^+^ and CD8^+^ T cell subsets are important for protection against this pathogen [22]. As the majority of microsporidian infections are acquired via the oral route, understanding the development of the gastrointestinal track immune response to these pathogens should be a focus of research and is critical in understanding how infection with these groups of organisms proceeds and in understanding the development of immune-based strategies to modulate the outcome of infection. Studies in this direction have demonstrated the induction of a strong and early intraepithelial lymphocyte (IEL) response to *E. cuniculi* infection (Figure 1) [22]. IEL are located in the lining of the gastrointestinal track epithelium and are commonly thought to represent one of the first lines of defense against orally acquired infections. This unique and very complex population of cells is comprised mainly of CD8^+^ T cells bearing CD8αβ TCRαβ, CD8ααTCRαβ, and TCRγδ CD8αα phenotype [23]. In studies conducted with *E. cuniculi*, the CD8αβ population was the most important cellular subset involved in protection against this pathogen, mediated by IFNγ production and the ability of these cells to exhibit cytotoxic activity against infected targets [24]. Moreover, adoptive transfer of these cells to immune-compromised mice partially protected these animals against lethal infection with *E. cuniculi*. The involvement of a cytotoxic response and IFNγ production by IELs in host protection was further confirmed by experiments using adoptive transfer of cells from IFNγ^−^ knockout and perforin knockout mice that proved unable to confer protection from infection in susceptible hosts.

The importance of IEL responses is not restricted to *E. cuniculi* infection and these cells have been reported to play a protective role against several other gastrointestinal pathogens. Studies in the past 30 years have demonstrated that IEL responses can be generated against pathogens like *Toxoplasma gondii*, rotavirus, simian immunodeficiency virus, *Listeria monocytogenes*, and *Giardia lamblia*, which are primarily acquired via a per-oral route. However, due to lengthy isolation techniques and poor cell recovery both in term of yield and quality, there is a scarcity of data about gastrointestinal immunity to oral pathogens in general, and many critical questions remain unanswered. One major issue is related to the factors or mechanism(s) involved in IEL priming, and studies in this direction are almost unavailable. We reported that IFNγ-producing DC from mucosal sites were critical for the generation of an optimal CD8αβ IEL response against *E. cuniculi* infection [24]; further studies in this direction need to be conducted and the class of mucosal DC involved in the priming and trafficking of these cells in response to *E. cuniculi* infection should be characterized in detail. While both chemokine receptors CCR2 and CCR5 have been implicated in the trafficking of IEL after *T. gondii* infection [25], [26], ongoing studies in our laboratory have recently demonstrated that IEL from CCR7-deficient mice are unable to mount an optimal response against infection, and this defect seems to be contingent on trafficking of infected DC from the gastrointestinal track to the draining lymph nodes (M. Moretto, I. Khan, unpublished data). Further studies with multiple gut pathogens, where IELs play an important role in protection, need to be performed to confirm if this is a general feature of gastrointestinal track infections or if it is specific to infection with *E. cuniculi*. This will enable us to understand the migration pattern of these cells, which apparently are important for combating orally transmitted pathogens such as *E. cuniculi*. Furthermore, the role of other chemokine receptors, which are expressed by IEL population [27], still needs to be investigated in the context of microsporidia infection.

## T Cell Memory and Immunity

Although IELs seem important in controlling acute infection, their ability to provide long-term protection against recurring and/or persistent exposure still needs to be characterized. Due to their anatomic location, generation and maintenance of long-term antigen-specific IEL would be highly beneficial to prevent re-infection against gastrointestinal pathogens. Earlier studies with *T. gondii* have reported that long-term survival of antigen-specific IELs is possible and that they can prevent re-infection of hosts when subjected to challenge with the same organism [28]. Thus, vaccines directed at targeting the development of memory IEL response against oral pathogens would be a logical and useful therapeutic strategy to prevent infections. However, the scenario is more complex than previously expected. In a recent study, it was shown that the markers for memory IEL grossly differ from those recently used for systemic T cell populations [29]. Attempts to identify markers specific for memory IEL need to be pursued, as this unusual and critical cell population is quite complex and is different from the traditional T cell populations and compartments studied to date. An additional problem with microsporidia is lack of information about CD8^+^ T cell epitopes, which makes it impossible to generate tetramers without which bona fide antigen-specific cells cannot be identified. However, this situation has shown some progress as the major polar tube protein (PTP1) has been reported to induce a significant antigen-specific systemic CD8^+^ T cell response [30] and potential epitopes have been identified. Attempts to generate tetramers based on this information are underway and efforts need to be put into identifying other antigens involved in the generation of this response. The outcome of these studies will be highly beneficial in understanding the mechanism(s) of IEL mediated protection against *E. cuniculi* infection, which can possibly be extended to other orally transmitted pathogenic protists.

## Summary

Microsporidiosis has been implicated in causing morbidity in immune-compromised and immune-competent individuals. As microsporidiosis is acquired via an oral route, gut-associated lymphoid tissues have been found to be critical for the initial control of this infection. Studies from our laboratories have demonstrated that IELs play a dominant role in this protection; however, the mechanisms involved in the trafficking of these cells and their role in long-term immunity remain to be elucidated.
